# Temperature Effect Separation of Structure Responses from Monitoring Data Using an Adaptive Bandwidth Filter Algorithm

**DOI:** 10.3390/ma17020465

**Published:** 2024-01-18

**Authors:** Anqing Hu, Gang Liu, Changjun Deng, Jun Luo

**Affiliations:** 1 China Railway Southwest Research Institute Co., Ltd., Chengdu 610031, China; xffish136@163.com (A.H.); ztxnydcj@163.com (C.D.); 2 School of Civil Engineering, Chongqing University, Chongqing 400045, China; 3 School of Civil Engineering and Architecture, Chongqing University of Science & Technology, Chongqing 401331, China; jluo@cqust.edu.cn

**Keywords:** damage detection, temperature effect, multi-scale analysis, adaptive bandwidth filter, statistical regression

## Abstract

Temperature is one of the most important factors significantly affecting damage detection performance in civil engineering. A new method called the Adaptive Bandwidth Filter Algorithm (ABFA) is proposed in this paper to separate the temperature effect from quasi-static long-term structural health monitoring data. The Adaptive Bandwidth Filter Algorithm (ABFA) is referred to as an algorithm of automatically adjusting the frequency bandwidth filter via the particle swarm optimization (PSO) algorithm. Considering the obvious multi-scale feature of the collected data of civil structure, the acquired time series are divided into different time scales (for example, day, month, year, etc.), and these scales in the frequency domain correspond to the center frequencies of the adaptive bandwidth filter. The temperature effect on structure responses across different time scales is thereafter explored by adaptively adjusting the frequency bandwidth of the filter based on the known center frequencies of different scales. The relationship between the temperature and the structure responses is established through statistical regression facilitated by sufficient in situ monitoring data. Simulation and experiment results show the very promising performance of the proposed algorithm and decouple the temperature effect accurately from the contaminated data; thus an enhanced capability of damage detection is achieved.

## 1. Introduction

In the last few years, the static measurement of changes in structural responses has become an important method in the field of health monitoring of civil engineering structures, as a number of structural properties such as mass and stiffness directly influence structural responses. For most applications, the measured quantities are typically displacements or strains, and no matter what physical quantities are measured, the data acquisition process is subject to changing environmental and operational conditions. For continuous evaluation of the actual health conditions of structures, only long periods of in situ observations seem to give trustable and sustainable information. Due to the fact that measurements are obtained during long-term day-to-day operation, the collected data are contaminated by all types of variability from a huge amount of sources, including changes in environmental conditions, loading, slow concrete shrinkage and inevitable testing noise and errors. As a matter of fact, field tests have suggested that the influence of changing environmental conditions contributes the most, which is often larger than or comparable to the effect of structural damages in structural responses [1,2,3,4]. As a result, structural damage cannot be reliably identified directly using long-term collected signals without appropriate processing.

For example, among all the possible environmental factors that affect structural responses significantly, temperature is a very important one being actively investigated [5,6]. Farrar et al. [6] conducted vibration tests on the I-40 Bridge by cutting one of the bridge girders at four damage levels and concluded that damage could not be detected directly through the measured frequencies because the ambient temperature induces a large variation in the bridge’s dynamic characteristics. 

When multiple environmental parameters are involved, several kinds of regression and interpolation methods have been used to separate environmental influences through statistical models. The first type of statistical methods is to use linear models for regression such as static linear regression, which can be directly used to relate measured characteristics to the corresponding environmental conditions [7,8]. Sohn et al. [9] also adopt a linear filter between temperature inputs and frequency to capture the variation of the frequency response to temperature. The second type of statistical method is to use a dynamic or nonlinear mode for regression [10,11,12], such as the Auto-Regressive output and eXogenous input method (ARX) [10] and the principal component analysis method [11]. Other methods include building a regression model after decomposing the signals, such as wavelet transform [13,14,15]. When a new signal is reconstructed from the wavelet coefficients which correspond to the temperature effect, regression and interpolation methods may be employed to separate temperature influence. 

There are also methods proposed to eliminate the influence of changing environmental conditions in the case when temperature is not measured [16,17,18,19,20,21,22,23]. The singular value decomposition method and the auto-associative neural network method have been applied to implicitly model the underlying relationship between environmental variables and damage-sensitive features. Fritzen et al. [16] adopted the subspace-based identification method for temperature compensation. For extracting features not only sensitive to damage but also insensitive to environmental variations from the collected signals, Manson et al. [17] conducted principal component analysis to project the original feature space onto a reduced feature space, and identified damage by means of novel detection methods. Changxi Yang and Yang Liu [18] adopted principal component analysis to eliminate the environmental effects on natural frequencies considering nonlinearity. William Soo Lon Wah and Yining Xia [19] proposed a method to eliminate the effects of outlier measurements based on a multiple-regression model and smaller levels of damage have been identified. Yansong Diao et al. [20] conducted singular spectrum analysis to transform a measured frequency sequence into several independent components, and the impact of variable environmental situations can be eliminated by re-selecting suitable components. Numerical simulation and experiment are used to validate the proposed method.

Despite the many methods available to separate temperature effect, there are other factors that will affect long-term measurements and cause poor separation of temperature effects, such as changing environmental conditions, loading, slow concrete shrinkage and inevitable testing errors. The procedure of the recent method includes the following: firstly, daily and yearly scales are used for temperature, and the frequency of changes in temperature are obtained. Secondly, based on the fact that the center frequencies of day temperature and year temperature are fixed in the frequency domain, the precision of the temperate separation algorithm depends on the selected frequency bounds of each filter group. However, the frequency bounds are difficult to choose manually due to the influences of other factors, such as testing errors. Therefore, the particle swarm optimization (PSO) algorithm [24,25,26] is adopted to adjust the filter frequency bandwidths of different temperature types adaptively to separate the temperature effect more accurately. Finally, an Adaptive Bandwidth Filter Algorithm (ABFA) is proposed to decouple the temperature effect from continuous static monitoring data of civil structures in this work.

The paper is organized as follows: a review of algorithms used to eliminate or separate temperature-induced changes in structural responses is summarized in Section 1. The ABFA is discussed in detail in Section 2. In Section 3, the ABFA is applied to simulate data with testing noise. The results of the application are shown in Section 4, and Section 5 summarizes the paper and draws conclusions are at the end.

## 2. Algorithm of the Adaptive Bandwidth Filter

### 2.1. Model and Algorithm

During the long-term health monitoring processes, changes in structural static response $\delta_{D}(t)$ and temperature signal $\delta_{T}(t)$ can be treated as combinations of the following effects:

(1) $$\delta_{D}(t)=\delta_{DT}(t)+\delta_{DP}(t)+\delta_{DC}(t)+\delta_{DD}(t)+\delta_{DR}(t)\;$$

(2) $$\delta_{T}(t)=\delta_{TD}(t)+\delta_{TF}(t)\;+\delta_{TY}(t)\;$$

where $\delta_{DT}(t)$ is the response change caused by temperature, named the temperature effect (TE); $\delta_{DP}(t)$ is the loading effect; $\delta_{DC}(t)$ is the slow shrinkage of concrete effect; $\delta_{DD}(t)$ is the structural damage effect; and $\delta_{DR}(t)$ is the testing error effect. $\delta_{TD}(t)$ is the day temperature changes; $\delta_{TF}(t)$ is an abrupt temperature drop; and $\delta_{TY}(t)$ is the year temperature changes. Therefore, the temperature effects corresponding to day temperature effect, abrupt temperature drop and year temperature effect can be named DTE, ATE and YTE, respectively. Moreover, the temperature of a structure can be described by global temperature and section temperature. The global temperature represents the overall temperature change of the structure. The section temperature represents the different temperatures at different positions on the cross-section of the structural component. Therefore, the day temperature effect can be divided further into the global day temperature effect (GDTE) and the section day temperature effect (SDTE).

**Figure 1:**
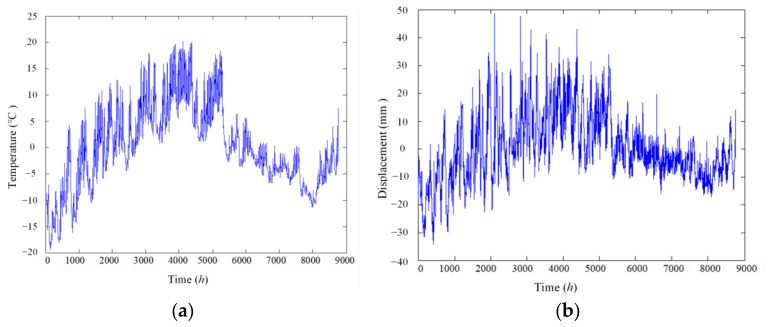
Static-based signal in time history collected in one year. (**a**) Temperature; (**b**) displacement.

**Figure 2:**
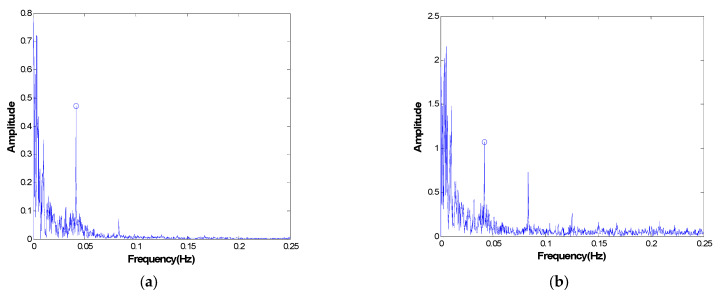
The amplitude spectrum of the collected signal in Figure 1. (**a**) Temperature; (**b**) displacement.

From Equations (1) and (2), it can be seen that a collected static-state signal is a combination of numerous types of different effects; thus it is impossible to directly separate temperature effect from any measured signals. For example, Figure 1 shows the environmental temperature and deflection of a bridge in China for one year sampled once every two hours, and the corresponding power spectra as functions of frequency are plotted in Figure 2. The correlation coefficient between temperature and displacement is evaluated to be greater than 0.87, indicating that the temperature is a major environmental factor influencing the structural static condition. However, there are many other factors also contributing to non-damage-induced changes in structures, which ought to be filtered as well. Due to the multi-scale characteristics of the static-state monitoring data [17], the temperature effect is not coupled with other effects in the same time scale; thus it is possible to use multi-scale analysis techniques to extract signals whose time scales are corresponding to $\delta_{TD}(t)$ , $\delta_{TF}(t)$ and $\delta_{TY}(t)$ , respectively, to regress temperature effect features more accuracy. Furthermore, damage-induced structural static response changes can be determined using low-pass filtering and the established temperature effect features.

To extract signals on a different time scale, the collected temperature and structural response data are filtered using band-pass filter groups, and therefore the design of the filters becomes the critical point to determine the precision of the decoupled signal. Thus, the problem can be expressed as selecting the optimal bandwidths of the filter groups to achieve the maximum accuracy of the separated signals. Due to the aforementioned non-linear influences of other factors except for temperature, choosing the bandwidths becomes a tough problem, and a well-designed optimization and parameter updating process is necessary. Because DTE is caused by sunrise and sunset every day and YTE is due to seasonal change, center frequencies of these two temperature effects can be determinate at the beginning, as shown in Figure 2.

According to the linear relationship between temperature and temperature-caused response in linear elastic structural deformation when a structure is subject to only slow temperature variation, the linear coefficient between them is used as the criterion to decide the extraction precision; that is, the objective function of the optimization process is the minimum error of temperature and structure response filtered using filter groups. Thus, the fitness function is expressed as:

(3) $$\underset{\left\{a,b\right\}}{\arg\min}\{\sum_{j=1}^{k}\sum_{i=1}^{n}\left|Y_{y}^{i}-({\hat{a}}_{yj}T_{yj}^{i}+{\hat{b}}_{yj})\right|+\sum_{j=1}^{k}\sum_{i=1}^{n}\left|Y_{d}^{i}-({\hat{a}}_{dj}T_{dj}^{i}+{\hat{b}}_{dj})\right|\}$$

where subscripts *y* and *d* represent the filter groups corresponding to day temperature effect (DTE) and year temperature effect (YTE); *k* is the total number of temperature sensors; *n* is the total number of collected signals; *Y* and *T* are the filtered displacement and temperature signal, respectively, obtained from taking the measured displacement and temperature signal passing through the two filter groups; ${\hat{a}}_{y}=\left\{{\hat{a}}_{y1},{\hat{a}}_{y2},\cdots ,{\hat{a}}_{yk}\right\},{\hat{b}}_{y}=\left\{{\hat{b}}_{y1},{\hat{b}}_{y2},\cdots ,{\hat{b}}_{yk}\right\}$ are the regression coefficients of *Y*; ${\hat{a}}_{d}=\left\{{\hat{a}}_{d1},{\hat{a}}_{d2},\cdots ,{\hat{a}}_{dk}\right\},{\hat{b}}_{d}=\left\{{\hat{b}}_{d1},{\hat{b}}_{d2},\cdots ,{\hat{b}}_{dk}\right\}$ are the regression coefficients of *T*; and |•| represents the absolute value. If there are too many temperature sensors in the same bridge, the quantity of regression coefficients ${\hat{a}}_{y},{\hat{b}}_{y},{\hat{a}}_{d},{\hat{b}}_{d}$ is quite large. In order to avoid ill-conditioned numerical problems in the regression, the principal component analysis technique should be conducted to reduce the number of temperature data [11].

Because DTE is caused by sunrise and sunset every day, daily temperature changes are more intense and the center frequency of DTE is higher. Similarly, because YTE is due to seasonal change, yearly temperature changes are slower and the center frequency of YTE is lower. Because the center frequency of YTE and DTE can be determined beforehand, the upper and lower frequency bounds of each filter group are employed as updating parameters. To satisfy the physics, parameter updating is constrained in the following range:

(4) $$\left\{\begin{array}{l} 0\leq f_{l}^{y}<f_{c}^{y},\\ f_{c}^{y}<f_{u}^{y}<f_{c}^{d}\\ f_{c}^{y}<f_{l}^{d}<f_{c}^{d}\\ f_{c}^{d}<f_{u}^{d}<f^{d} \end{array}\right.$$

where superscript *y* and *d* represent year and day filter groups, respectively; subscripts *l*, *c* and *u* are the pass, center and stop frequencies, respectively; and $f^{d}$ is the upper frequency of the day filter group and is valued as 1 in this work.

In conclusion, the rationality of frequency bounds of each filter group directly affects the estimation accuracy of regression coefficients. In order to automatically adjust the frequency bandwidth filter, particle swarm optimization (PSO) is introduced. The PSO algorithm was originally proposed by Kennedy and Eberhart as a simulation method of social behaviors. A detailed description on the technology is given in Refs. [24,25,26] and only a brief outline is presented here for clarity of explanation.

Initially, a swarm of individuals known as particles is randomly established and each individual represents an updating parameter. Its performance is evaluated through a predefined fitness function, and then the population is evolved into the next generation through the repositioning of its individuals. In the evolution from one generation to the next, the swarm is updated by the following equations:

(5) $$\left\{\begin{array}{l} v_{id}(t+1)=wv_{id}(t)+c_{1}r_{1}(p_{id}-z_{id}(t))+c_{2}r_{2}(p_{gd}-z_{id}(t))\\ z_{id}(t+1)=z_{id}(t)+v_{id}(t+1) \end{array}\right.$$

where vid represents the velocity of the *i*th particle in the *d*th dimension, $v_{id}\in [-v_{\max},v_{\max}]$ ; *v_max_* is the maximum velocity, which is a non-negative constant; *t* is an iteration counter; *r*_1_ and *r*_2_ are two random numbers in [0, 1]; *w* is the inertia weight; *c*_1_ and *c*_2_ are acceleration coefficients; *p_id_* and *p_gd_* are the best positions encountered by the ith particle and the whole swarm population so far in the *d_th_* dimension, respectively; and *z_i_*(*t*) represents the current position of particle *i*, while *z_i_*(*t* + 1) represents the updated position.

**Figure 3:**
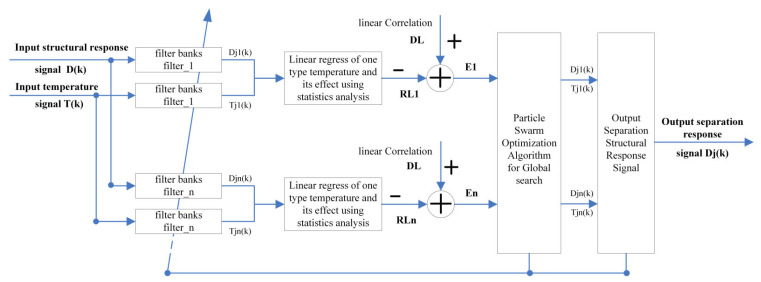
Principle of Adaptive Bandwidth Filter based on PSO.

An ABFA is proposed to adjust the filter frequency bandwidth adaptively using the PSO algorithm, demonstrated in Figure 3. After the original structural response signal *D*(*k*) and temperature signal *T*(*k*) are acquired from a structure, the filtered signal *D_j_*_1_(*k*) to *D_jn_*(*k*) and *T_j_*_1_(*k*) to *T_jn_*(*k*) are obtained from original signals with band-pass filter groups whose bandwidths are chosen arbitrarily corresponding to different temperature effects. As to each temperature effect, the residue is calculated with Equation (3) between the filtered signal and the statistics analysis. Then the bandwidth of each filter group is modified by the PSO algorithm iteratively until the optimization termination condition is satisfied. The separation response signal *D_j_*(*k*) with the maximum precision is obtained by the summation of regression models of different temperatures. The procedure of the ABFA is as follows:

1. Obtain the same time span signal of temperature and structure response.
2. Set the center frequencies of DTE and YTE.
3. Set the iteration index t = 0 and filter updating parameters $f_{l}^{i}$ and $f_{u}^{i}$ (*i* = d and y, corresponding to DTE and YTE, respectively) randomly.
4. Compute the cost function according to Equation (3). Update the particle velocities, position *f_i_*, and local and global memories according to Formula (5).
5. Limited new particle positions to lie within a logical bound according to Formula (4) so that the band-pass filter can work.
6. If *t* < *t_max_* (the maximum cycle time), then *t* = *t* +1 and go to step 4; otherwise stop iteration.
7. Use the best *f_i_* obtained from PSO to filter the signals of temperature and structure response so that two different time scale signals corresponding to DTE and YTE are acquired.
8. Regress linear factor of DTE and YTE to day temperature (DT) and year temperature (YT), respectively.
9. Calculate day and year temperature effects by summation of linear factor multiplied by DT and YT, respectively.

### 2.2. Performance Measures

**Figure 4:**
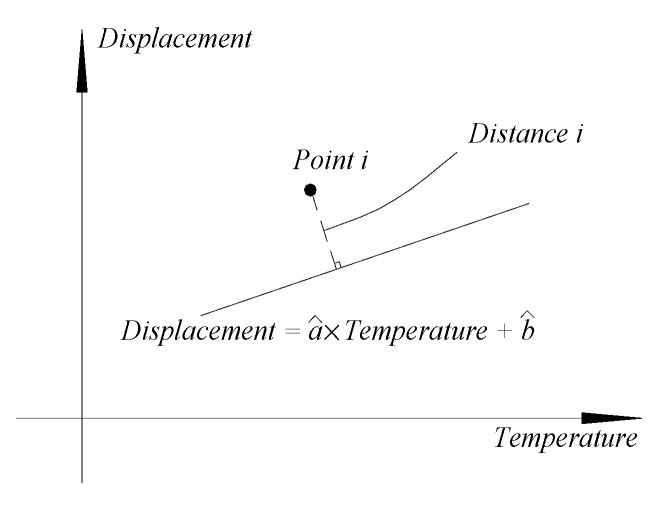
Distance of a point to the regression line.

An integrated assessment index (*IAI*) was introduced to evaluate the performance of the ABFA. The *IAI* is defined as follows:

(6) IAI=DF×CCF

where *DF* is used to describe the average distance from the filtered signal to the regression line shown in Figure 4, and is given by:

(7) $$DF=\sum_{i=1}^{Nm}d_{i}/Nm$$

where *N_m_* is the number of collected signals and *d_i_* is the distance of the *i_th_* filtered signal to the regression line in Figure 4.

The correlation coefficient factor (*CCF*) between temperature and the residual signal is defined as:

*CCF* = |*Corrcoef*(residual displacement, temperature)| = |cov(X,Y)/(σ_X_∗σ_Y_)|

where *corrcoef* is the function in Matlab, cov(X,Y) is the covariance of X and Y, and σ_X_ is the standard deviation of X.

If the linear relationship between temperature and response is eliminated, the residual signal is no longer linear with temperature, and CCF will decrease. Meanwhile, if the trend of displacement with temperature change is fitted more accurately, smaller deviations in DF will be obtained. Therefore, the index IAI will decrease when temperature effects can be more accurately estimated and eliminated. Obviously, the smaller the value of IAI is, the better ABFA performs.

## 3. Simulation Analysis

In order to illustrate the proposed ABFA-based approach for temperature separation and damage diagnosis under varying environmental conditions, an example is deployed in this section. The performance of both temperature separation and damage diagnosis is considered in the simulation section. The day temperature difference and year temperature difference are considered in the temperature signal. The daily global temperature difference effect, daily section temperature difference effect, year temperature difference effect and the slow shrinkage and creep effect are considered in the displacement signal. And then, the performance of temperature separation is discussed. Furthermore, instantaneous and cumulative damages are considered, and the damages are identified using the residual displacement signal after removing the temperature effect.

### 3.1. The Simulated Temperature and Displacement Signals

The simulated temperature signal is considered to be composed of the day temperature difference and the year temperature difference. The amplitude of the day temperature difference is assumed to be 5 °C, and the year temperature difference is 30 °C. Since the day temperature difference and year temperature difference are periodic, they are herein assumed to be in a cosine pattern. Thus the daily temperature difference *T*_1_ and year temperature difference *T*_2_ can be expressed as

(8) $$\left\{\begin{array}{l} T_{1}\left(t\right)=2.5\times \cos\left(\pi t/12\right)\\ T_{2}\left(t\right)=15\times \cos\left(\pi t/4380\right) \end{array}\right.$$

The simulated displacement signal is considered to be composed of two kinds of displacements, i.e., the displacements caused by various temperature differences and slow shrinkage and creep. The daily global temperature difference effect *f*_1_, daily section temperature difference effect *f*_2_, year temperature difference effect *f*_3_ and the slow shrinkage and creep effect *f*_4_ versus time can be obtained using the following formulae:

(9) $$\left\{\begin{array}{l} f_{1}\left(t\right)=0.1\times \cos\left(\pi t/12\right)\\ f_{2}\left(t\right)=0.02\times \cos\left(\pi t/12\right)\\ f_{3}\left(t\right)=0.6\times \cos\left(\pi t/4380\right)\\ f_{4}(t)=-1.45\times {\left(t-300/12000+(t-300)\right)}^{0.65} \end{array}\right.$$

Therefore, the formulae of temperature and displacement are as follows:

(10) $$T_{0}(t)=T_{1}(t)+T_{2}(t)$$

(11) $$f_{0}(t)=f_{1}(t)+f_{2}(t)+f_{3}(t)+f_{4}(t)$$

**Figure 5:**
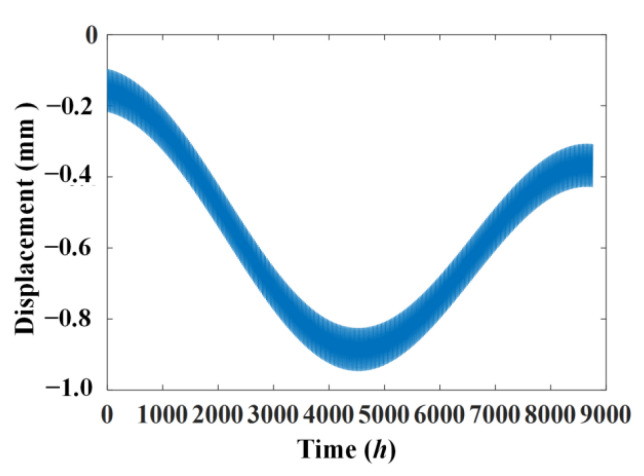
Simulated displacement signal.

**Figure 6:**
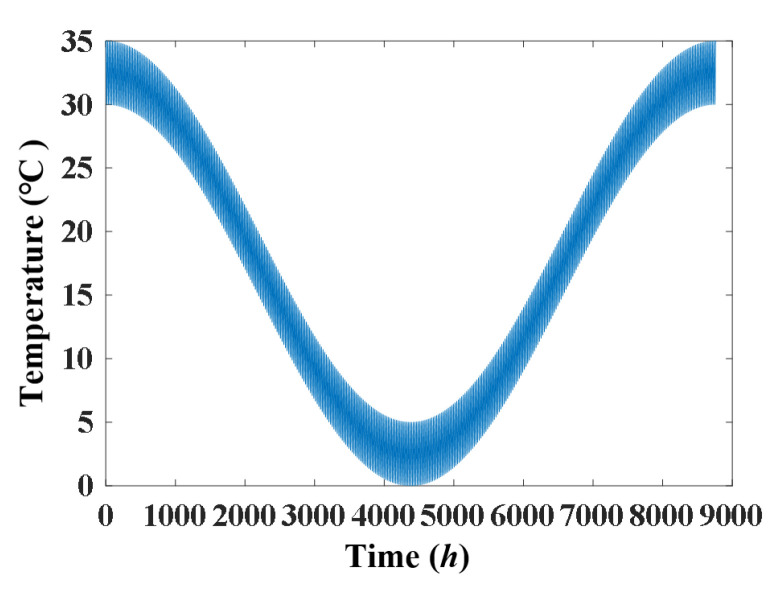
Simulated temperature signal.

**Figure 7:**
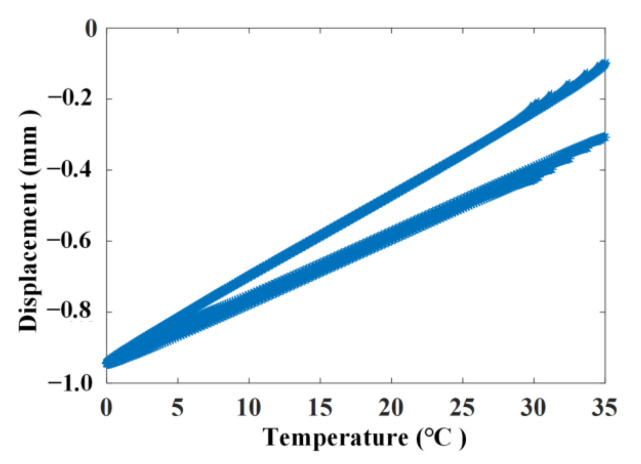
Linear correlation of the simulating signal without noise.

The simulated displacement and temperature signals according to Formulae (10) and (11) are shown in Figure 5 and Figure 6, for which the simulated time is 8760 h, equal to one year. Figure 7 shows the linear correlation between them. The results show that there is an increasing trend of displacement versus temperature and the fork is due to the slow shrinkage and creep effect.

**Figure 8:**
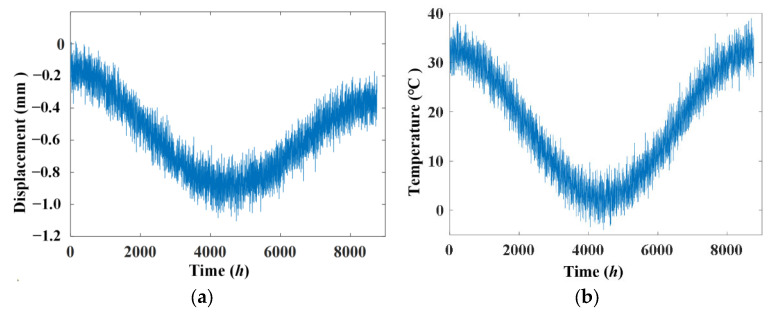
Simulated displacement signal and temperature signal with noise. (**a**) Displacement signal; (**b**) temperature signal.

**Figure 9:**
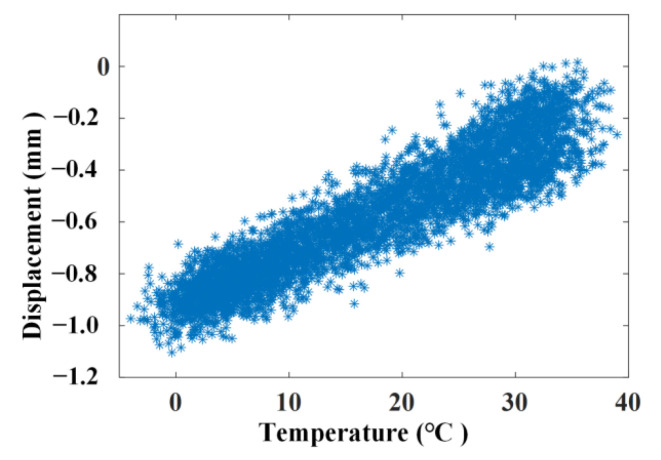
Linear correlation of the simulating signal with noise.

To study the efficiency of the ABFA under testing noise, the displacement signal *f*_0_(*t*) is perturbed by adding testing noise, and the obtained signal *f*(*t*) with testing noise is expressed as:

(12) $$\begin{array}{l} f(t)=f_{0}(t)+\sum_{i=100}^{140}0.05\times rand\times (\sin(10\pi t/i))\\ T(t)=T_{0}(t)+\sum_{i=100}^{140}1.5\times rand\times (\sin(10\pi t/i)) \end{array}$$

where *rand* is a random number in the interval [0, 1]. The simulated displacement signal and temperature signal according to Equation (12) are shown in Figure 8 and the linear correlation between displacement and temperature with noise is shown in Figure 9. The results show that noise has a significant impact on the simulated displacement signal and the weaker effect of slow shrinkage and creep has been masked. Only the various temperature effects have been displayed and an increasing trend of displacement versus temperature is obtained.

### 3.2. Relationship between Linear Correlation Using the ABFA

Using Butterworth band-pass filters, the center frequency of DTE is set to be 1/24 (taking hours as seconds). The maximum attenuation of the pass band is 0.1 dB, and the minimum attenuation of the hinder band is 30 db. The particle number is 40, and every 5 particles compose a particle swarm to carry out a local search. The maximum iteration is 30, c_1_ and c_2_ are 2.0, and the max velocity V_max_ is 5.

**Figure 10:**
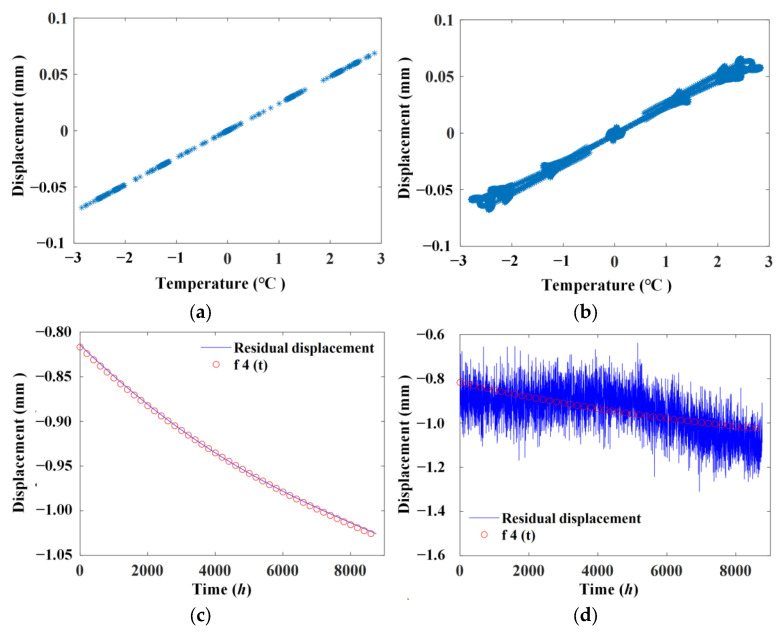
Linear correlation and residual displacement signal after extraction using ABFA. (**a**) Without noise; (**b**) with noise; (**c**) residual displacement signal after removing temperature effect without noise; (**d**) residual displacement signal after removing temperature effect with noise.

Figure 10a shows the linear correlation after extraction using the ABFA without noise, and Figure 10b illustrates the results with noise. Figure 10c shows the residual displacement signal after removing the temperature effect using the ABFA without noise, and Figure 10d illustrates the results with noise.

It can be seen from Figure 10 that with the use of the ABFA, the linear correlation between temperature and temperature-caused displacement is explicitly illustrated, which indicates that effects other than temperature are effectively eliminated. Furthermore, the residual displacement signal after removing the temperature effect without noise and the signal f4(t) match well. With noise, the residual displacement signal after removing the temperature effect gradually decreases as time increases, which has a similar trend of change as the signal f4(t).

### 3.3. Relationship between Linear Correlation Using the Long Short-Term Memory Network (LSTM) Method

**Figure 11:**
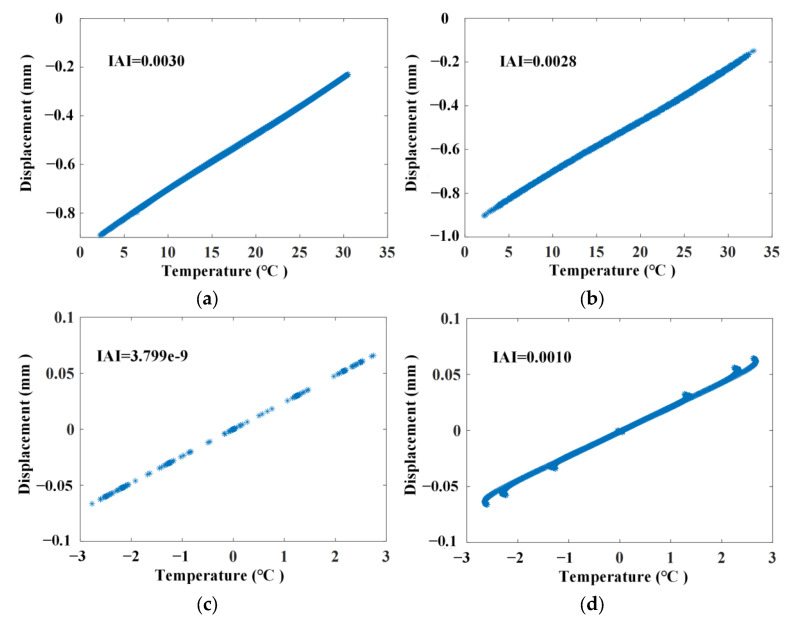
Linear correlation after extraction using LSTM and ABFA. (**a**) Without noise using LSTM; (**b**) with noise using LSTM; (**c**) without noise using ABFA; (**d**) with noise using ABFA.

The long short-term memory network (LSTM) method is used to compare how temperature affects separation. The number of network layers for this LSTM is set to 2. The input dimension and output dimension are both 2, and the hidden layer has 128 neurons. The first 60% of displacement and temperature signals are selected as the training set, and the last 40% signals are selected as the test set. Based on the results from the test set, Figure 11a shows the linear correlation after extraction using LSTM without noise, and Figure 11b illustrates the results with noise. And the corresponding *IAI* values can be calculated and marked in the diagrams.

Meanwhile, based on the results from the last 40% signal displacement and temperature signals extracted using ABFA, a linear correlation can be drawn, which is shown in Figure 11c,d. And the corresponding *IAI* values can be calculated and marked in the diagrams. The results in Figure 11 show that the LSTM and ABFA methods can both be used to separate the temperature effects. However, the ABFA method has smaller *IAI* values, because the accuracy of separation will decrease as the data length of the test set increases in the LSTM method.

### 3.4. Relationship between IAI and Different Time Spans of Signals Using the ABFA

The *IAI* values of different time spans with or without noise are listed in Table 1. It can be seen that the *IAI* value decreases as the time span increases. Comparing the results of three years with those of six months, the *IAI* value decreases from 2.09 × 10^−5^ to 3.07 × 10^−8^ without noise; whereas in the condition with noise, the IAI value decreases from 0.0050 to 0.0014. Therefore, the longer the collected time is, the better the performance of the ABFA will be, especially when the collected signals are perturbed with noise. This can be attributed to two reasons: Firstly, as the time span increases, the frequency resolution will increase correspondingly so that the frequency bands beside the temperature effect, such as the random component as shown in Formula (12), can be filtered out. Secondly, in the process of regression, the stochastic factor will be cut down with more time.

However, the classification of temperature into day temperature and year temperature is merely in time scale. As for the influence of temperature on structural response, there is no difference. Therefore, using both YTE and DTE or only DTE for regression of the temperature effect can fully describe the influence of temperature.

With the time span increasing from one year to three years, the *IAI* produces small changes, as shown in Table 1, but calculation time increases rapidly. So it is advised to use day temperature and DTE to separate the temperature effect only if there are few year period data acquired. The advantages of using DTE instead of DTE and YTE simultaneously in evaluating the performance of separating temperature effect are as follows

1. The computation time can be cut down greatly.
2. A reliable health assessment on the bridge can be advanced. Actually, in order to meet the requirement of frequency resolution for the year temperature filter, several year reference periods are needed at least. But for most bridges, especially for deteriorated bridges, this period is so long that collapse accidents may not be prevented timely.

### 3.5. Damage Detection Using the ABFA

**Figure 12:**
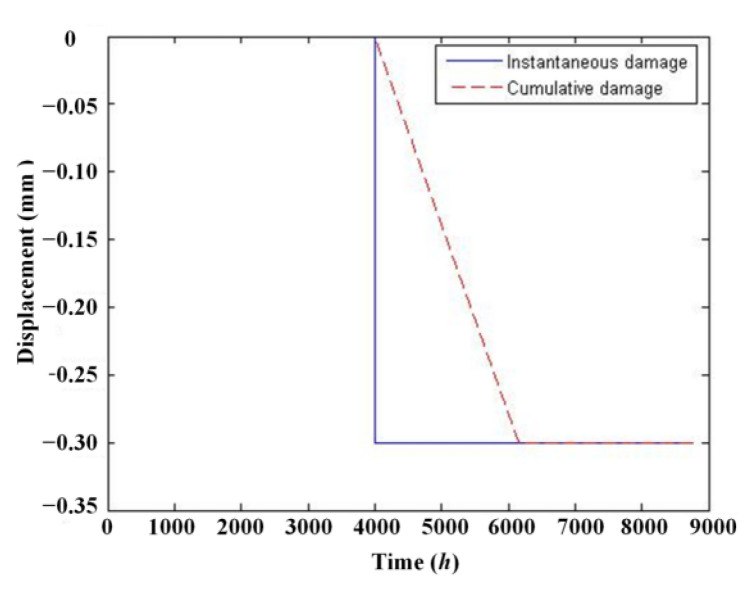
The damage case.

Damage will be detected more easily with the temperature effect identified and eliminated from the data. In most cases, damage falls into two groups: instantaneous damage caused by extreme events, such as earthquakes and impact from ships, as well as cumulative damage which results from a long deterioration process. For the sake of brevity, only two damage cases, as shown in Figure 12, will be adopted and only one-year time span data are utilized. The damage case time history is added to another time history according to Formula (12), and the temperature data are simulated with Formula (12).

**Figure 13:**
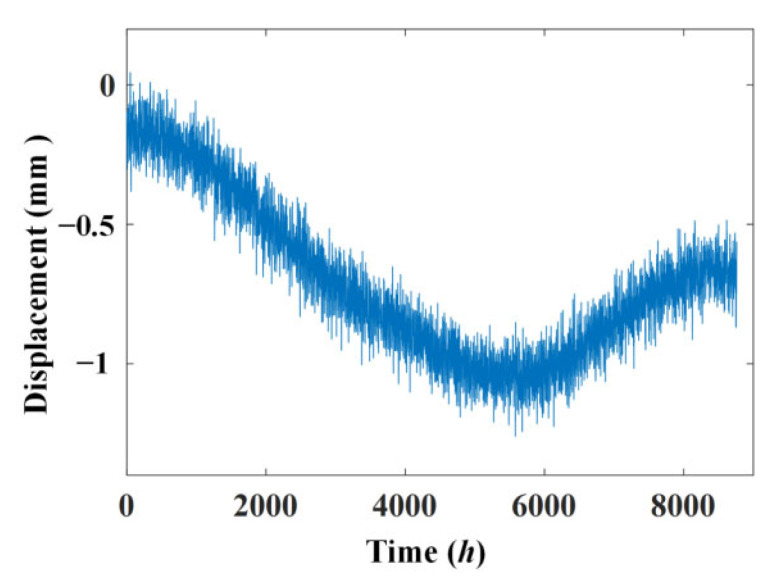
Simulated displacement signal with noise and cumulative damage.

**Figure 14:**
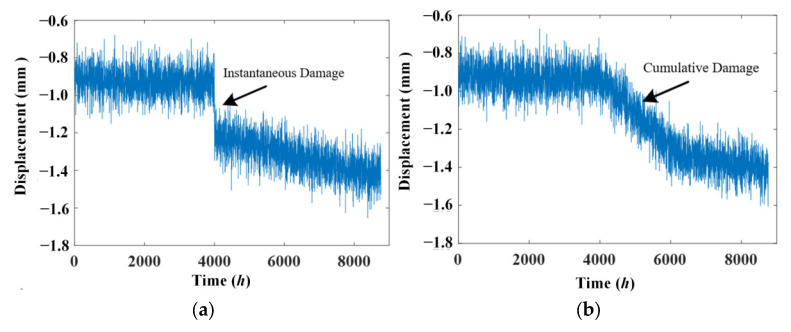
Residual displacement signal after removing temperature effect. (**a**) Instantaneous damage case; (**b**) cumulative damage case.

Figure 13 indicate that damage cannot be detected from the original signal because there is no evident diversification. Residual displacement signals after removing the temperature effect using ABFA and PSO are shown in Figure 14.

In either the instantaneous or cumulative damage case, the residual signals indicate that there are some different trends in the displacement signal at the 4000 h location. The damage can be detected more easily than from original signals. The damage in both cases can be detected successfully because the frequency of damage is not in the frequency domain of day or year temperature effect. And this phenomenon also exists in real bridges.

## 4. Applications

Signals collected from an arch bridge reinforced with concrete-filled steel tubes built in China, which has existed for ten years, are adopted to verify the ABFA. Because man-made damage is infeasible for real-world bridges, the performance of the damage diagnosis cannot be explored in the experimental section; only the temperature separation results are discussed in this section.

### 4.1. The Measured Temperature and Displacement Signals

**Figure 15:**
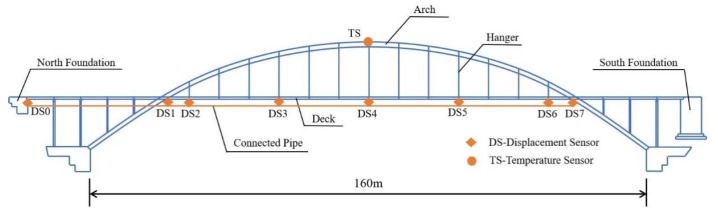
The elevation view and part sensor location of the monitored bridge.

The main span of the bridge is 160 m long and the distance between each hanger is 8 m. Experiments are conducted on the bridge to measure changes in vertical displacement and temperature at the surface of the mid-span. The locations and arrangements of the sensors of the health monitoring system installed in this bridge are shown in Figure 15. In total, eight sensor locations were selected to measure the desired signal, in which DS0 at the north foundation was the base point and DS1 to DS7 were the monitoring points. At each displacement sensor location, a connected pipe optoelectronic liquid level sensor was installed under the deck of the bridge to collect the vertical displacement. And a temperature sensor was mounted at the bottom of the mid-span arch.

**Figure 16:**
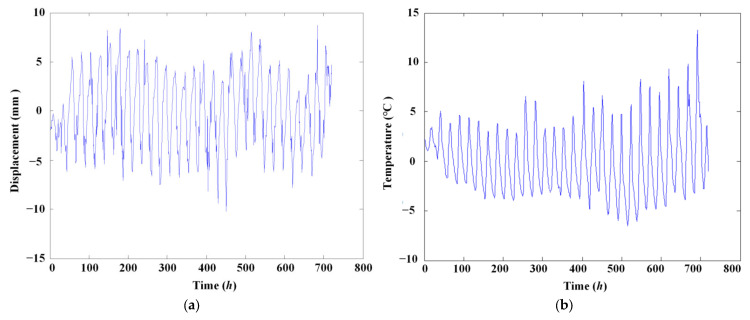
Measured signals from experimental bridge. (**a**) Temperature signal; (**b**) displacement signal.

**Figure 17:**
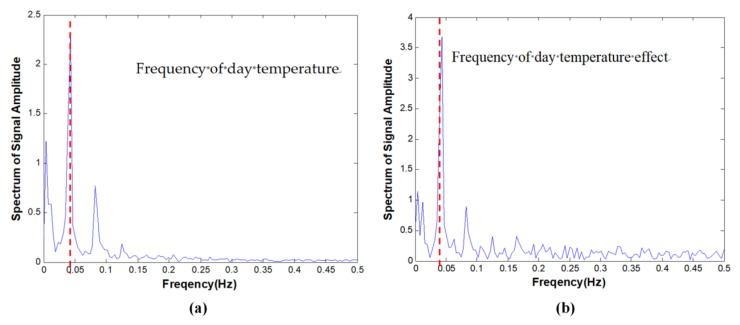
Spectrum amplitude. (**a**) Temperature; (**b**) displacement.

The measured temperature and displacement variation signals from temperature sensor TS and displacement sensor DS4 located at mid-span over one month are shown in Figure 16. The displacement sensor is a static level. The amplitude spectra are shown in Figure 17.

### 4.2. Relationship between Linear Correlation Using the ABFA

**Figure 18:**
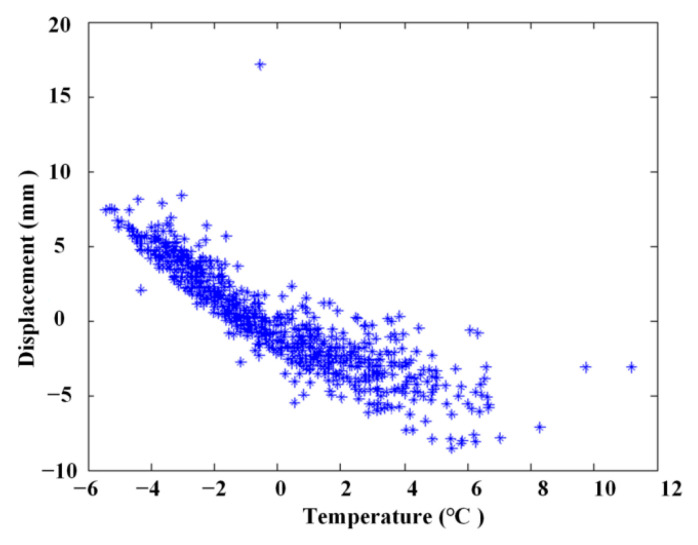
Linear correlation of measured signals.

**Figure 19:**
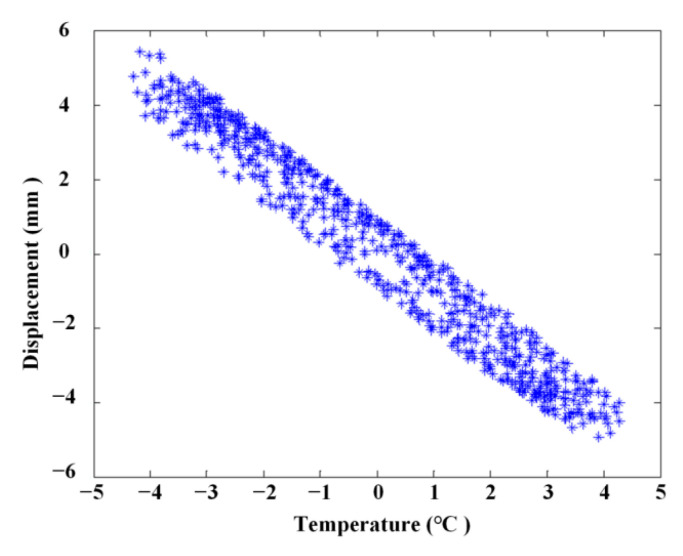
Linear correlation after extraction using PSO.

The linear relationship of measured temperature and displacement (Figure 16) is illustrated in Figure 18, and the linear relationship after extraction using the ABFA is shown in Figure 19. It is obvious that the linear correlation between measured temperature and displacement is attenuated due to the influences of other factors, as shown in Figure 18. In this case, linear statistical regression will be incapable of reaching an accurate relationship between temperature and displacement. Figure 19 shows that the linear correlation between measured temperature and displacement is largely improved after extraction using the ABFA.

### 4.3. The Temperature Separation Results Using ABFA

**Figure 20:**
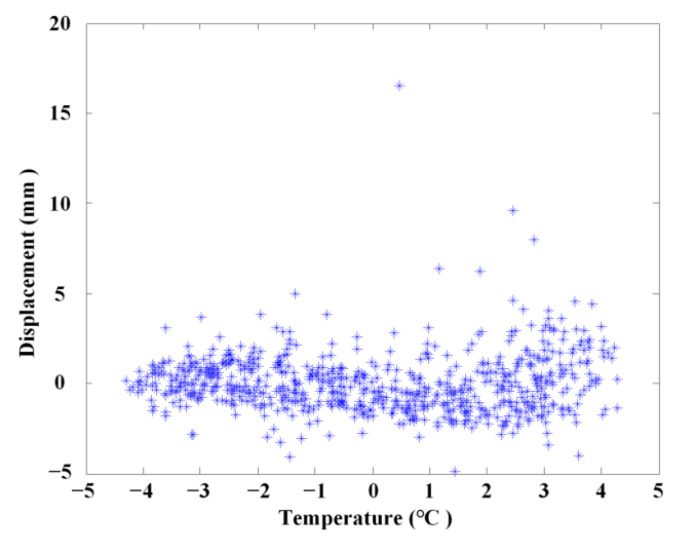
After removing temperature effect.

A more accurate relationship between temperature and displacement is obtained using linear statistical regression. The linear correction of vertical displacement with temperature after separation of the temperature effect is demonstrated in Figure 20. It can be seen from Figure 20 that the residual displacement signal after removing the temperature effect mainly corresponds to the vehicle load and measurement error as the linear coefficient between the residual displacement signal and temperature is nearly 0. The results show that the proposed ABFA method can indicate temperature influence, and effectively eliminates the influence of temperature on the measurement signal.

## 5. Conclusions

A new method (ABFA), which is proposed as a temperature effect determination algorithm for the continuous static-based monitoring of civil engineering structures, is presented in this paper based on the PSO algorithm. Also, an index (IAI) is defined to assess the performance of different parameters in the ABFA. The proposed method can automatically adjust the frequency bandwidth filter, reduce the interference from testing errors on manually selecting filter parameters and better eliminate the impact of temperature. A simulation example is used to illustrate the proposed ABFA-based approach for data cleansing under varying environmental conditions, including temperature changes and testing noise. The results show that the temperature effect can be effectively detected and eliminated using the proposed method, and the damage can be detected more easily. The results of experimental data have also shown that the temperature effect can be more accurately determined by using the ABFA than directly extracting from the original collected signals. Although this method depends on the availability of various temperature parameters, fortunately, data of these parameters are usually accessible seeing that temperature sensors are installed in most long-term structural health monitoring systems.

## Figures and Tables

**Table 1:** Performance comparison of different time scales based on *IAI* index.

	Six Months	One Year	Two Years	Three Years
No noise	2.09 × 10^−5^	1.60 × 10^−7^	6.06 × 10^−8^	3.07 × 10^−8^
With noise	0.0050	0.0043	0.0026	0.0014

## Data Availability

Data are contained within the article.

## References

[1] Wah W.S.L. (2023). Damage detection of structures under changing environmental and operational conditions using the COVRATIO statistic. Eng. Struct..

[2] Luo J., Liu G., Huang Z., Law S. (2019). Mode shape identification based on Gabor transform and singular value decomposition under uncorrelated colored noise excitation. Mech. Syst. Signal Process..

[3] Lei Z., Liu G., Cong Y., Tang W. (2024). Research on fatigue damage mitigation of offshore wind turbines by a bi-directional PSTMD under stochastic wind-wave actions. Eng. Struct..

[4] Liu G., Li M., Mao Z. (2020). Dynamic monitoring of structure in civil engineering for phase motion estimation via Hilbert transform. Mech. Syst. Signal Process..

[5] Gaebler K.O., Hedegaard B.D., Shield C.K., Linderman L.E. (2018). Signal selection and analysis methodology of long-term vibration data from the i-35w st. anthony falls bridge. Struct. Control. Health Monit..

[6] Farrar C.R., Baker W.E., Bell T.M., Cone K.M., Darling T.W., Duffey T.A., Eklund A., Migliori A. (1994). Dynamic Characterization and Damage Detection in the I-40 Bridge over the Rio Grande.

[7] Standoli G., Clementi F., Gentile C., Lenci S. (2023). Post-earthquake continuous dynamic monitoring of the twin belfries of the Cathedral of Santa Maria Annunziata of Camerino, Italy. Procedia Struct. Integr..

[8] Maes K., Meerbeeck L.V., Reynders E.P.B., Lombaert G. (2022). Validation of vibration-based structural health monitoring on retrofitted railway bridge KW51. Mech. Syst. Signal Process..

[9] Sohn H., Dzwonczyk M., Straser E.G., Kiremidjian A.S., Law K.H., Meng T. (1999). An experimental study of temperature effect on modal parameters of the Alamosa Canyon Bridge. Earthq. Eng. Struct. Dyn..

[10] Zimin V.D., Zimmerman D.C. (2009). Structural Damage Detection Using Time Domain Periodogram Analysis. Struct. Health Monit..

[11] Datteo A., Luca F., Busca G. (2017). Statistical pattern recognition approach for long-time monitoring of the g.meazza stadium by means of AR models and PCA. Eng. Struct..

[12] Wang J., Tong X., Yue C., Liu W., Zhang Q., Zeng L., Huang G. (2023). Real-time temperature distribution reconstruction via linear parameter-varying state-space model and Kalman filter in rack-based cooling data centers. Build. Environ..

[13] Shah S.A.A., Bais A., Alashaikh A., Alanazi E. (2023). Discrete wavelet transform based branched deep hybrid network for environmental noise classification. Comput. Intell..

[14] Eltotongy A., Awad M.I., Maged S.A., Onsy A. Fault Detection and Classification of Machinery Bearing Under Variable Operating Conditions Based on Wavelet Transform and CNN. Proceedings of the 2021 International Mobile, Intelligent, and Ubiquitous Computing Conference: International Mobile, Intelligent, and Ubiquitous Computing Conference (MIUCC).

[15] Yu X., Liang Z., Wang Y., Yin H., Liu X., Yu W., Huang Y. (2022). A wavelet packet transform-based deep feature transfer learning method for bearing fault diagnosis under different working conditions. Measurement.

[16] Fritzen C.R., Mengelkamp G., Guemes A. (2003). Elimination of temperature effects on damage detection within a smart structure concept. Struct. Health Monit..

[17] Manson G. Identifying Damage Sensitive, Environmental Insensitive Features for Damage Detection. Proceedings of the Third International Conference Identification in Engineering Systems, University of Wales Swansea.

[18] Yang C., Liu Y. (2021). Detecting the damage of bridges under changing environmental conditions using the characteristics of the nonlinear narrow dimension of damage features. Mech. Syst. Signal Process..

[19] Soo Lon Wah W., Xia Y. (2022). Elimination of outlier measurements for damage detection of structures under changing environmental conditions. Struct. Health Monit..

[20] Diao Y., Sui Z., Guo K. (2021). Structural damage identification under variable environmental/operational conditions based on singular spectrum analysis and statistical control chart. Struct. Control. Health Monit..

[21] García-Macías E., Hernández-González I.A., Puertas E., Gallego R., Castro-Triguero R., Ubertini F. (2022). Meta-Model Assisted Continuous Vibration-Based Damage Identification of a Historical Rammed Earth Tower in the Alhambra Complex. Int. J. Archit. Herit..

[22] Jabid Q., Luis M., Rodolfo V., Magda R., Johanatan C. (2017). PCA based stress monitoring of cylindrical specimens using pzts and guided waves. Sensors.

[23] Tenelema F.J., Delgadillo R.M., Casas J.R. (2022). Bridge Damage Detection and Quantification under Environmental Effects by Principal Component Analysis.

[24] Khanesar M.A., Yan M., Isa M.A., Piano S., Branson D.T. (2023). Precision Denavit–Hartenberg Parameter Calibration for Industrial Robots Using a Laser Tracker System and Intelligent Optimization Approaches. Sensors.

[25] Chafi M.R.S., Narm H.G., Kalat A.A. (2023). Calibration of fluxgate sensor using Least Square Method and Particle Swarm Optimization Algorithm. J. Magn. Magn. Mater..

[26] Mhaya A.M., Baghban M.H., Faridmehr I., Huseien G.F., Abidin A.R.Z., Ismail M. (2021). Performance Evaluation of Modified Rubberized Concrete Exposed to Aggressive Environments. Materials.

